# Flexural Response of Dense Polymeric BCC Lattice Beams: Experimental Benchmark and Limits of Homogenized Beam Descriptions

**DOI:** 10.3390/polym18162003

**Published:** 2026-08-17

**Authors:** Gastón Sal-Anglada, Marta Moure Cuadrado, Javier Paz, Matías Braun

**Affiliations:** 1 Barcelona Supercomputing Center—Centro Nacional de Supercomputación (BSC–CNS), 08034 Barcelona, Spain; 2 Aerospace Systems and Transport Research Group, Rey Juan Carlos University, 28933 Madrid, Spain; marta.moure@urjc.es (M.M.C.); javier.paz@urjc.es (J.P.); 3 Research Group GREEN, University of Nebrija (UAN), 28015 Madrid, Spain; mbraun1@nebrija.es

**Keywords:** BCC lattice, bending stiffness, homogenized beam model, shear correction, strain-gradient elasticity, additive manufacturing

## Abstract

The flexural behaviour of body-centred cubic (BCC) lattice beams fabricated by stereolithography remains supported by limited experimental evidence, and available homogenized beam models are rarely confronted with data in the combined regime of high relative density, non-slender struts, and low span-to-depth ratios. This work presents an experimental campaign on polymeric BCC lattice beams with three unit-cell edge lengths (L=3, 4, and 5 mm) and a constant strut-to-cell ratio R/L=1/6, yielding a relative density $\rho^{*}\approx 0.423$. Specimens were tested under uniaxial compression and three-point bending for nine combinations of geometry. The experimental data are compared with three analytical frameworks: a classical Euler–Bernoulli homogenized beam model and a BCC-specific shear-corrected formulation at the structural level, both evaluated without calibration to the bending tests, together with a strain-gradient extension whose intrinsic length scale is calibrated against them. For the effective Young’s modulus, the closed-form expression of Lee et al. reproduces the compression data within 10%, whereas the Tancogne-Dejean and Mohr model remains markedly stiffer even after the strut-level Timoshenko correction is included. In bending, none of the models proves adequate over the full geometric range: the Euler–Bernoulli model is accurate for several configurations (errors below 16% in five of nine cases) but overestimates the stiffness by up to 108% for the deepest specimen; the shear-corrected model reduces the global root mean square error from 97.94 to 24.44 N/mm (approximately a factor of four), but introduces excessive flexibility in some slender and intermediate configurations; and the strain-gradient correction, being strictly stiffening, yields no appreciable improvement. To avoid assigning the discrepancy to a single mechanism, the bending data are further analysed through an experimental compliance decomposition. The additional compliance relative to Euler–Bernoulli theory is small or negative in several cases, showing that shear flexibility alone cannot explain the full dataset, but becomes dominant for the deepest beams. The results therefore delineate the range of validity of simple homogenized beam models for dense finite BCC lattice structures and identify the combined influence of structural shear, non-slender struts, nodal-region morphology, finite-cell and boundary effects, local roller-contact compliance, and the discrete distribution of struts across the cross-section as the main mechanisms requiring more refined descriptions. These findings correspond to a single relative density ($\rho^{*}\approx 0.423$) and a single strut-to-cell ratio (R/L=1/6), so the resulting span-to-depth indicator ($L_{0}/h\approx 2.5$) should be regarded as indicative for this class of dense lattices rather than as a general design rule.

## 1. Introduction

In recent years, advances in additive manufacturing techniques have allowed the development of metamaterials based on lattice structures, which would be unfeasible to obtain using traditional fabrication methods [1,2,3]. Such structures have high potential for industrial applications because their effective mechanical properties can be tailored through the geometry, connectivity, and dimensions of the underlying architecture. Modifications to parameters such as the position, length, and diameter of the bars facilitate the attainment of design-specific properties. This results in architectured materials with favourable strength-to-weight ratios, high energy absorption capacity, and directional stiffness control [4,5]. These characteristics make lattice structures promising candidates as cores of sandwich structures [6,7,8,9], including in aerospace applications [10], where mass reduction and mechanical efficiency are important design drivers [11].

In light of the growing interest in these metamaterials, it is crucial to develop tools that facilitate their design optimization. A substantial body of research has been dedicated to the development of numerical tools for the prediction of the mechanical behaviour of metamaterials based on lattice structures [12,13,14,15]. These models, based on the finite element method, have been demonstrated to accurately predict the behaviour of these metamaterials, with results validated against experimental data.

Although finite element models are an invaluable design tool, the generation of numerical models during the design process can be a laborious and time-consuming process. It is therefore important to develop analytical models that facilitate the design of these metamaterials, not only to optimize the design process but also to enhance comprehension of the fundamental physics underlying these materials.

A substantial body of research has been dedicated to the development of analytical models for the design of metamaterials based on fundamental lattice structures [16,17,18,19]. These models, based on either Euler–Bernoulli beam theory or Timoshenko beam theory, have successfully predicted the tensile and compressive stiffness of blocks composed of these metamaterials, demonstrating a high degree of correlation with experimental results.

As previously stated, one of the principal applications of these metamaterials is as the core of sandwich structures [20,21,22], which are frequently subjected to bending. It is therefore imperative to develop analytical models that can predict the bending stiffness of such structures in a simple and computationally inexpensive manner.

Recently, there has been a growing interest in developing analytical models to predict the bending stiffness of lattice metamaterials based on octet-truss structures. In the work presented by Korshunova et al. [23], a variety of models were introduced based on classical beam theory and strain-gradient theory. The capability of these models was then validated with experimental and numerical results. However, in their work, the application of the analytical models entailed the introduction of an equivalent elastic modulus, which was defined based on experimental or numerical results.

To address these issues, the objective of this study is not only to compare analytical predictions against bending experiments, but also to identify which parts of the discrepancy can and cannot be interpreted within simple homogenized beam descriptions. This distinction is important for dense finite lattices, for which the specimen may behave partly as an architectured material with effective properties and partly as a discrete structure governed by finite-cell geometry, boundary cells, nodal regions, and contact conditions. The bending rigidity is first predicted by combining an effective Young’s modulus obtained from unit-cell analytical models with a homogenized Euler–Bernoulli beam formulation. A shear-corrected homogenized beam model is then evaluated to represent the additional flexibility expected in deep beams with low span-to-depth ratios. Finally, a strain-gradient extension is considered as an additional correction introducing an intrinsic length scale parameter to account for possible size-dependent stiffening effects. To validate and critically assess these descriptions, experiments were conducted on resin specimens manufactured using low-force stereolithography, with three different unit-cell sizes considered. The experimental campaign includes both uniaxial compression tests of the resin material and the BCC structures, used to assess the accuracy of the effective Young’s modulus predictions, and three-point bending tests of the BCC structures, which constitute the primary validation dataset for the bending rigidity models. In addition to the direct stiffness comparison, an experimental compliance decomposition is introduced to quantify the apparent non-Euler–Bernoulli contribution without requiring additional numerical simulations.

## 2. Experimental Setup

### 2.1. Geometry and Material Characterization

The specimens employed in this study were produced using low-force stereolithography (LFS), with a Raise3D DF2 resin 3D printer (Raise3D, Irvine, CA, USA) and Apricot V1 resin (Raise3D, Irvine, CA, USA). Apricot V1 is a methacrylate-based photopolymer of the durable class; it was selected because it reproduces the sub-millimetre strut geometry with high fidelity while offering greater toughness than standard rigid resins, and because its behaviour under the present low-force stereolithography process had been characterised in previous work [3]. The printing parameters—such as layer thickness, fixed at 0.05 mm, build orientation, and support structures—were carefully selected to ensure dimensional accuracy and adequate mechanical performance. The specimens were sliced with ideaMaker v5.2.4.8581 (Raise3D, Irvine, CA, USA) using the manufacturer’s qualified high-detail template for the Apricot V1 resin, which is optimised for fine-feature fidelity; the 0.05 mm layer thickness balances resolution and build time. The complete set of printing and post-processing parameters is reported in Table 1, and the general characterisation framework for stereolithography materials follows [24]. All specimens were printed flat on the build platform, with the layer-deposition planes horizontal and coincident with the plane of the loaded faces, so that in both the compression and the three-point bending tests the load was applied perpendicular to the printing layers; this orientation was kept identical for all specimens, so that any process-induced anisotropy affects every configuration consistently. Because the homogenized beam models adopted here assume an isotropic effective medium, any residual anisotropy is one of the idealisations underlying the analytical predictions. After fabrication, all specimens were washed for 10 min and post-cured using a DF-Cure system, with each side exposed for 15 min and a heat-cure temperature of 60 °C under ambient conditions.

Dimensional accuracy was controlled during slicing through a calibrated in-plane scale compensation of 100.69% (in *X* and *Y*) and a contour offset of −0.054 mm for the Apricot V1 resin, which compensate for cure-induced shrinkage and edge light-bleed. The outer dimensions and the strut diameter of the as-fabricated specimens were verified after post-curing and agreed with the nominal values within the resolution of the measuring instrument, with no appreciable systematic deviation; the nominal strut-to-cell ratio R/L=1/6 is therefore representative of the fabricated specimens.

**Figure 1:**
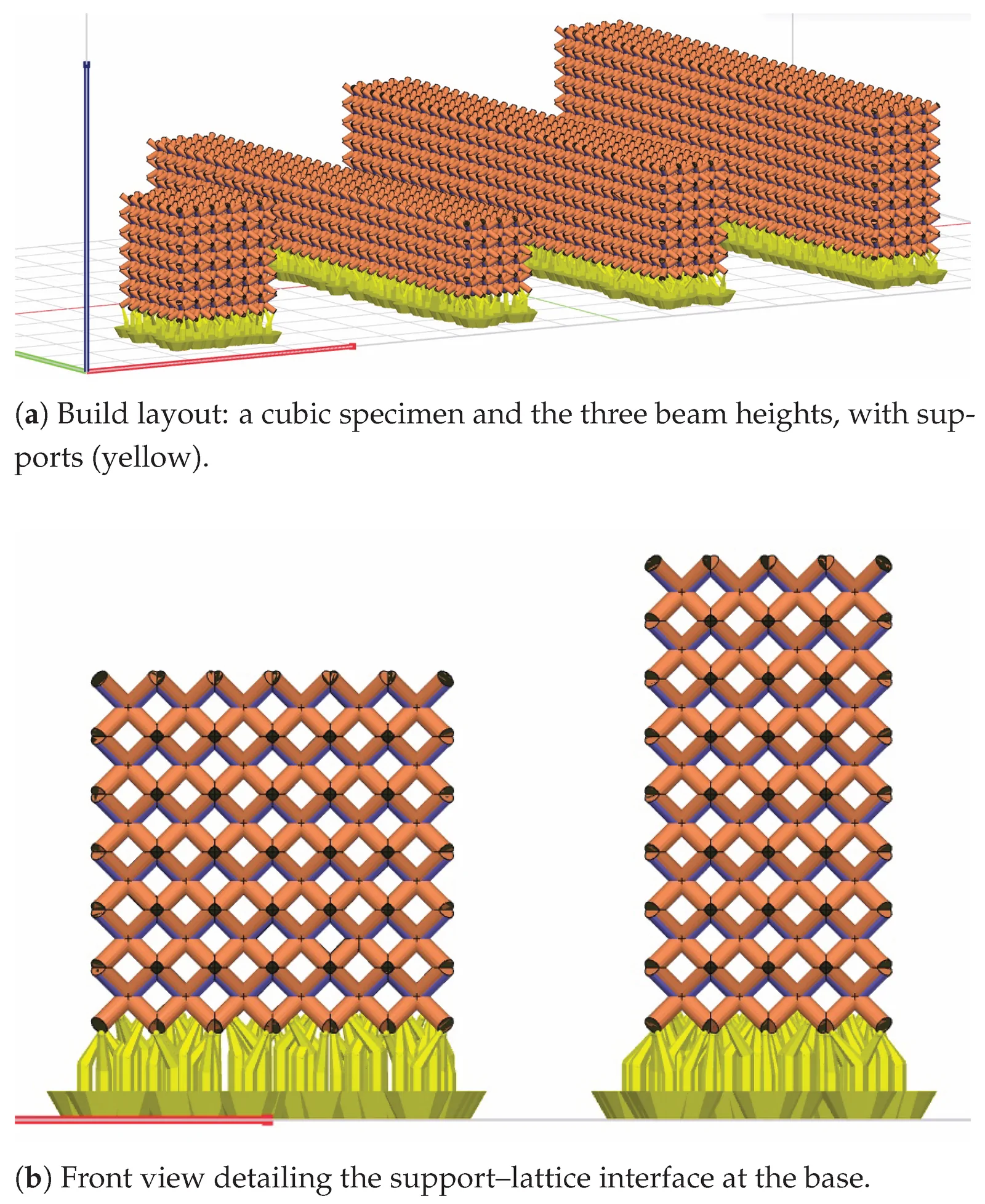
ideaMaker slicer previews showing the build layout and support strategy (touch-platform-only mode, $40^{\circ }$ overhang): supports (yellow) are generated only on the down-facing platform side. (**a**) perspective view of a cubic compression specimen together with the three bending-beam heights on the build plate; (**b**) front view detailing the support–lattice interface at the base. In the mechanical tests this support-contact face was placed as the upper (compression) face, leaving the lower tensile face free of support marks.

Support structures were generated automatically in the touch-platform-only mode, so that supports were created only on the down-facing side of the specimens during printing, with a $40^{\circ }$ overhang threshold, a support-point spacing of 2.5 mm (1.5 mm at edges) and a support-point contact offset of 0.35 mm. The supports were removed manually before post-curing. In the mechanical tests, the support-contact face was placed as the upper (compression) face of the beam, so that the lower tensile face—where three-point bending fracture was consistently observed to initiate at mid-span—remained free of support marks. The build layout and support strategy are shown in Figure 1.

In order to characterize the mechanical properties of the base material, uniaxial compression tests were performed on solid cylindrical specimens with a height of 30 mm and a diameter of 10 mm. The tests were conducted using a servo-hydraulic universal testing machine (Instron 8801, Instron, Norwood, MA, USA) with a 150 kN frame, at a constant displacement rate of 1 mm/min, controlled with Bluehill Universal v4.55 (Instron, Norwood, MA, USA). The load was applied to the specimens via 150 kN rated compression plates, thus minimizing the effect of machine compliance on the results. Load and displacement data were extracted from the 100 kN load cell and the LVDT equipped with the machine (Instron, Norwood, MA, USA). Both the specimen geometry and testing conditions were defined in accordance with ASTM D695.

**Figure 2:**
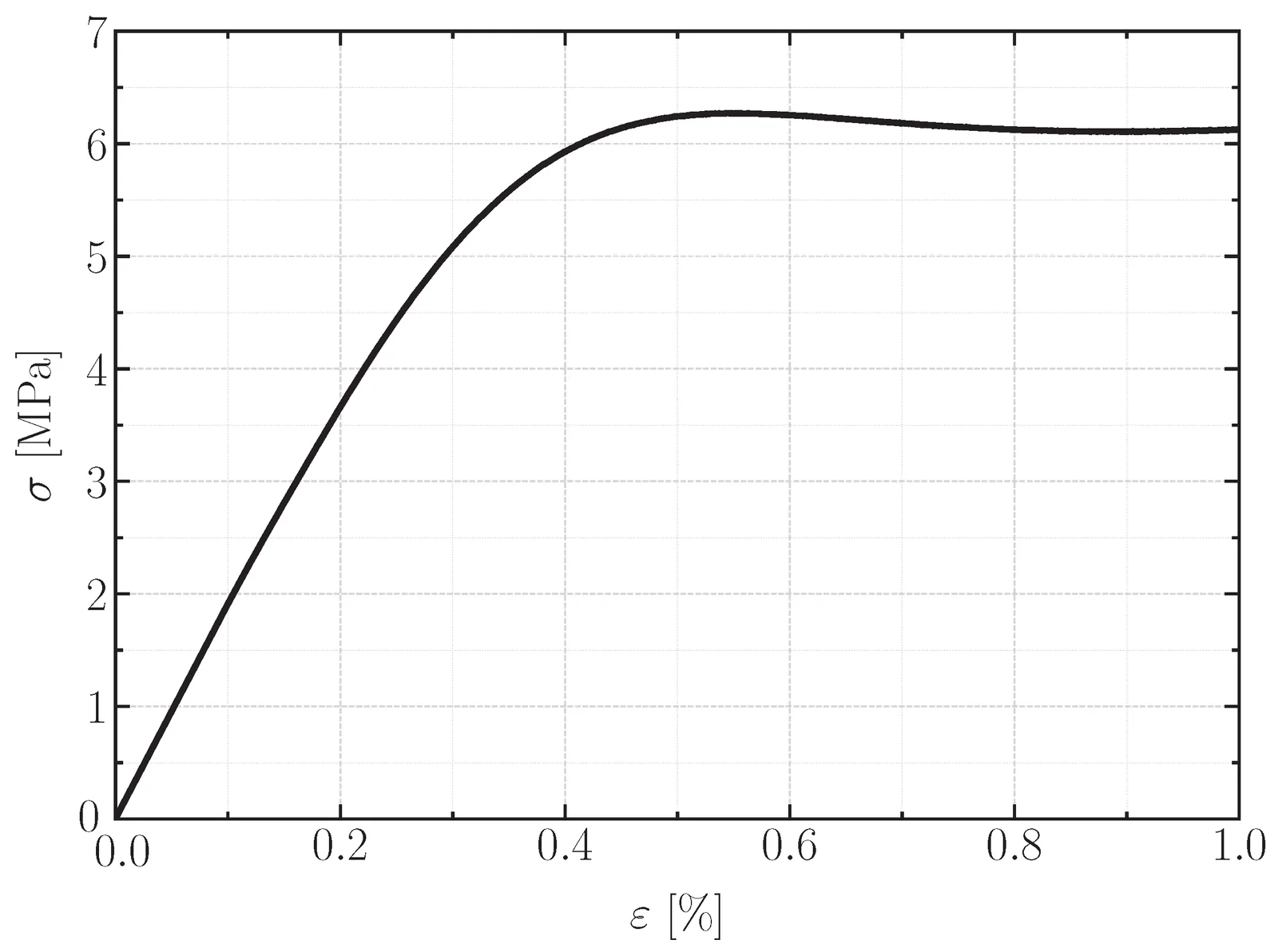
Representative stress–strain curve obtained from the compression tests on cylindrical resin specimens.

A total of three tests were carried out for the characterization of the resin’s elastic properties, and a representative stress–strain curve is presented in Figure 2. Based on these experiments, the Young’s modulus $E_{s}$ of the material was determined as 1724.7±78.1MPa (mean ± standard deviation; 1724.7±194 MPa at 95% confidence, Student’s *t*, n=3). The measured density of the resin was $1.40\;g/{\mathrm{cm}}^{3}$, in agreement with the manufacturer’s specifications. A Poisson’s ratio $\nu_{s}\approx 0.35$, representative of methacrylate-based photopolymers, was adopted where required.

**Figure 3:**
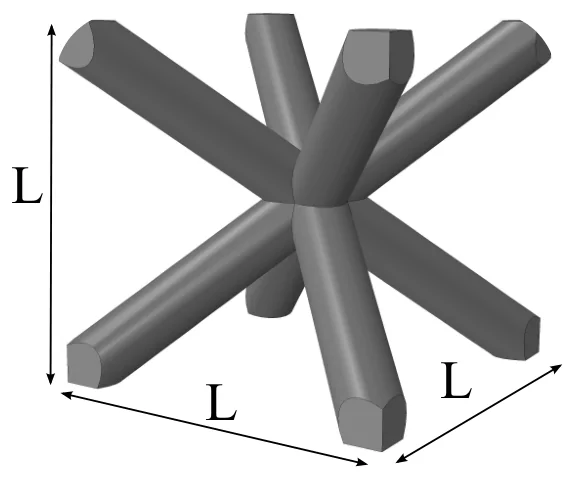
Configuration of the BCC unit cell.

The geometry and notation of the BCC unit cell are shown in Figure 3.

For specimens made of a single material with uniform circular struts of radius *R*, it is necessary to distinguish between the cubic unit-cell edge length, denoted here as *L*, and the center-to-vertex strut length, denoted as *ℓ*. For a BCC unit cell,

(1) $$\ell =\frac{\sqrt{3}}{2}L.$$

Following the geometrical convention used by Tancogne-Dejean and Mohr [25], the relative density of a BCC lattice made of uniform circular struts is expressed in terms of R/ℓ as

(2) $$\rho^{*}=3\sqrt{3}\pi {\left(\frac{R}{\ell }\right)}^{2}-18\sqrt{2}{\left(\frac{R}{\ell }\right)}^{3}.$$

This expression accounts for the volume of the struts and the nodal intersections. The beam intersections are referred to as nodes, whose volume fraction becomes increasingly important as the relative density increases. For example, Tancogne-Dejean and Mohr report that approximately 5% of the solid phase is contained in the nodes at $\rho^{*}=0.1$, whereas the nodal contribution increases to approximately 40% at $\rho^{*}=0.5$. Therefore, the present relative density, $\rho^{*}\approx 0.423$, places the specimens in a regime where the nodal regions and the non-slender character of the struts cannot be regarded as small perturbations. This point is retained throughout the discussion: the analytical strut-based expressions are used as compact homogenized estimates, but the observed bending response may also be affected by nodal morphology, finite-cell cross-sections, and boundary-cell effects.

Three cubic unit-cell edge lengths were considered, L=3, 4, and 5 mm. Rather than fixing the strut radius, the ratio between the strut radius and the unit-cell edge length was kept constant at

(3) $$\frac{R}{L}=\frac{1}{6}\approx 0.1667.$$

This resulted in nominal strut radii of R=0.500, 0.667, and 0.833 mm for L=3, 4, and 5 mm, respectively. Using Equation (1), these values correspond to

(4) $$\frac{R}{\ell }=\frac{2}{\sqrt{3}}\frac{R}{L}\approx 0.1925.$$

Substituting into Equation (2) gives an approximately constant relative density of $\rho^{*}\approx 0.423$ for all configurations.

### 2.2. Compression Tests

**Figure 4:**
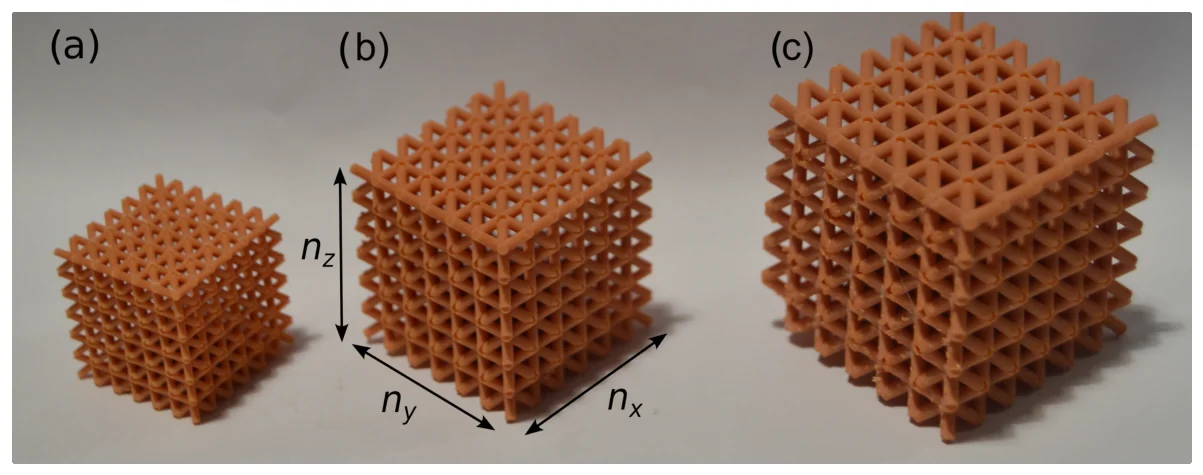
Geometry of the cubic BCC lattice specimens employed in the compression tests. All configurations are built from $n_{x}=n_{y}=n_{z}=6$ unit cells along each principal direction: (**a**) L=3 mm, overall dimensions 18×18×18 mm; (**b**) L=4 mm, overall dimensions 24×24×24 mm; (**c**) L=5 mm, overall dimensions 30×30×30 mm.

Uniaxial compression tests were performed on cubic lattice specimens to evaluate their effective mechanical response. Each specimen was designed with six unit cells along each spatial direction, resulting in overall dimensions of 18 mm, 24 mm and 30 mm for unit cell edge lengths of L=3, 4 and 5 mm, respectively, as shown in Figure 4.

The tests were conducted using the same testing machine and testing conditions as previously described for material characterization, under displacement control at a constant loading rate of 1 mm/min. For each configuration, three tests were performed to ensure repeatability.

**Figure 5:**
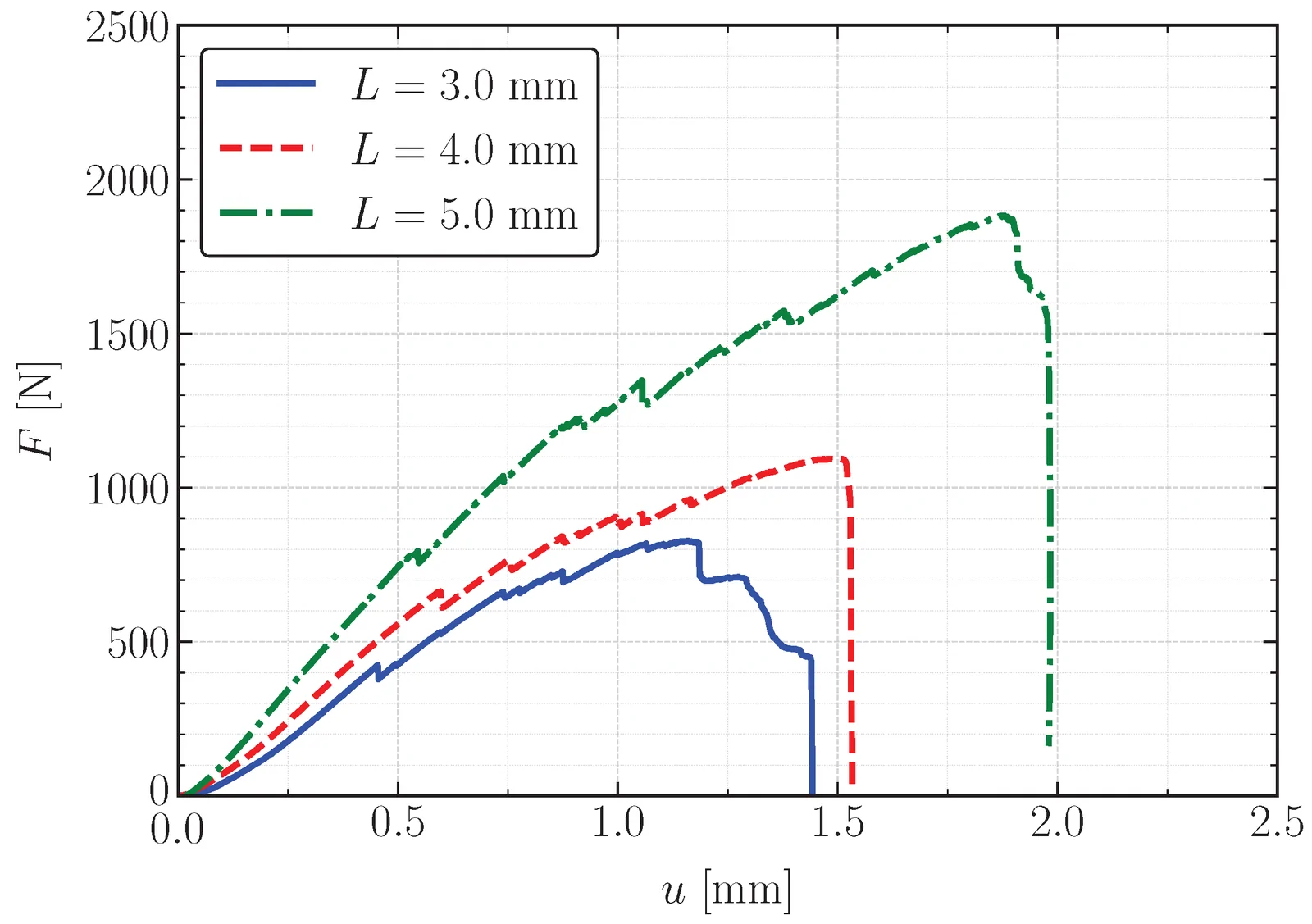
Representative force–displacement curves for cubic BCC lattice specimens with different unit-cell sizes.

From the obtained force–displacement curves, the initial stiffness of each specimen was determined. A representative force–displacement curve for each test sample size is presented in Figure 5. As can be observed, both the maximum load and the initial stiffness increase as the unit cell size increases, while maintaining a constant number of unit cells. These results provide a qualitative comparison of the mechanical response, whereas the corresponding quantitative values are discussed in conjunction with the analytical predictions in the Results section.

### 2.3. Bending Tests

**Figure 6:**
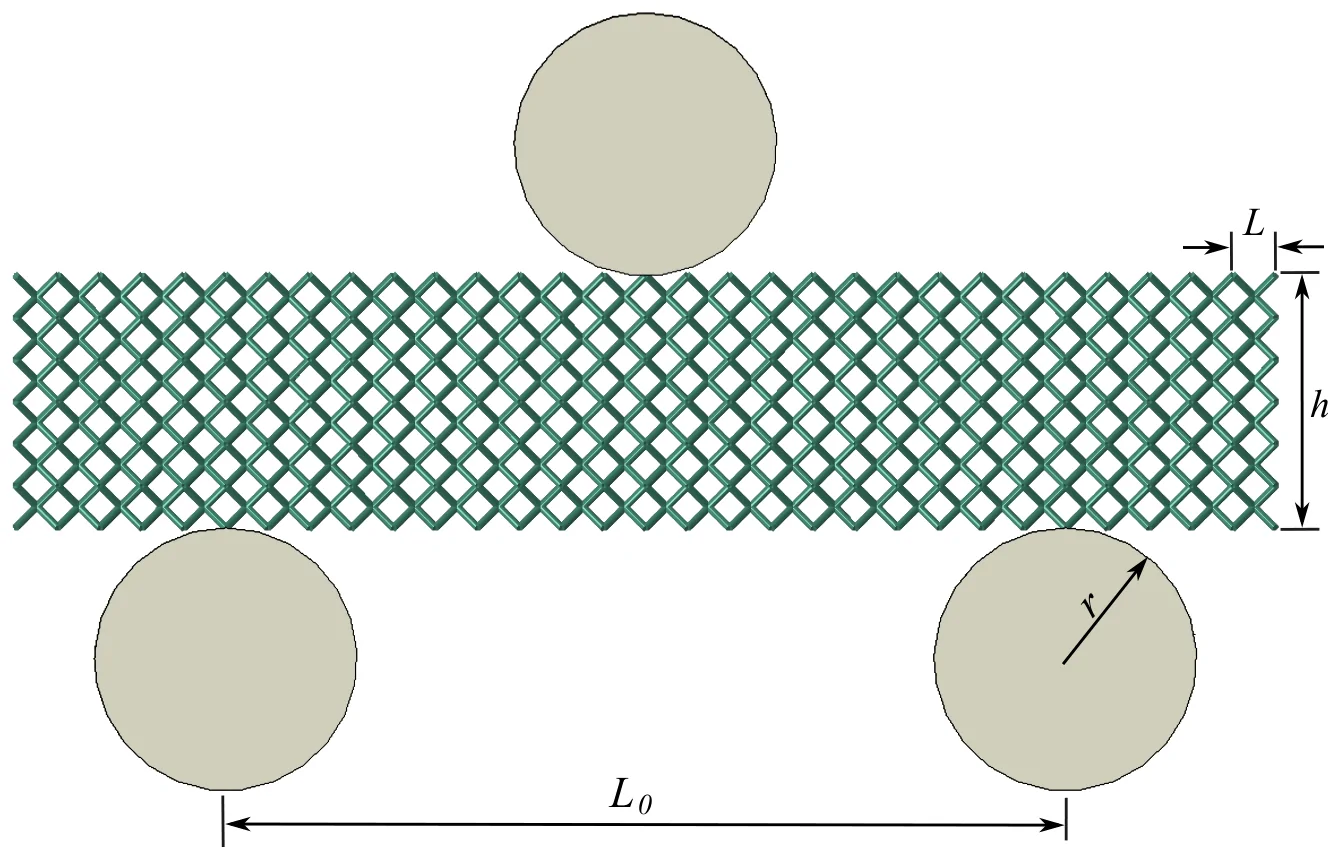
Schematic representation of the three-point bending test configuration. The specimen, of total length 100 mm, rests on two cylindrical supports separated by a span of $L_{0}=80$ mm, while the load *F* is applied at mid-span through a third cylindrical roller. The bottom rollers displace upwards while the upper roller stays stationary. All loading and support rollers have a radius of r=12.5 mm. The vertical displacement *u* at the loading point is measured during the test.

**Figure 7:**
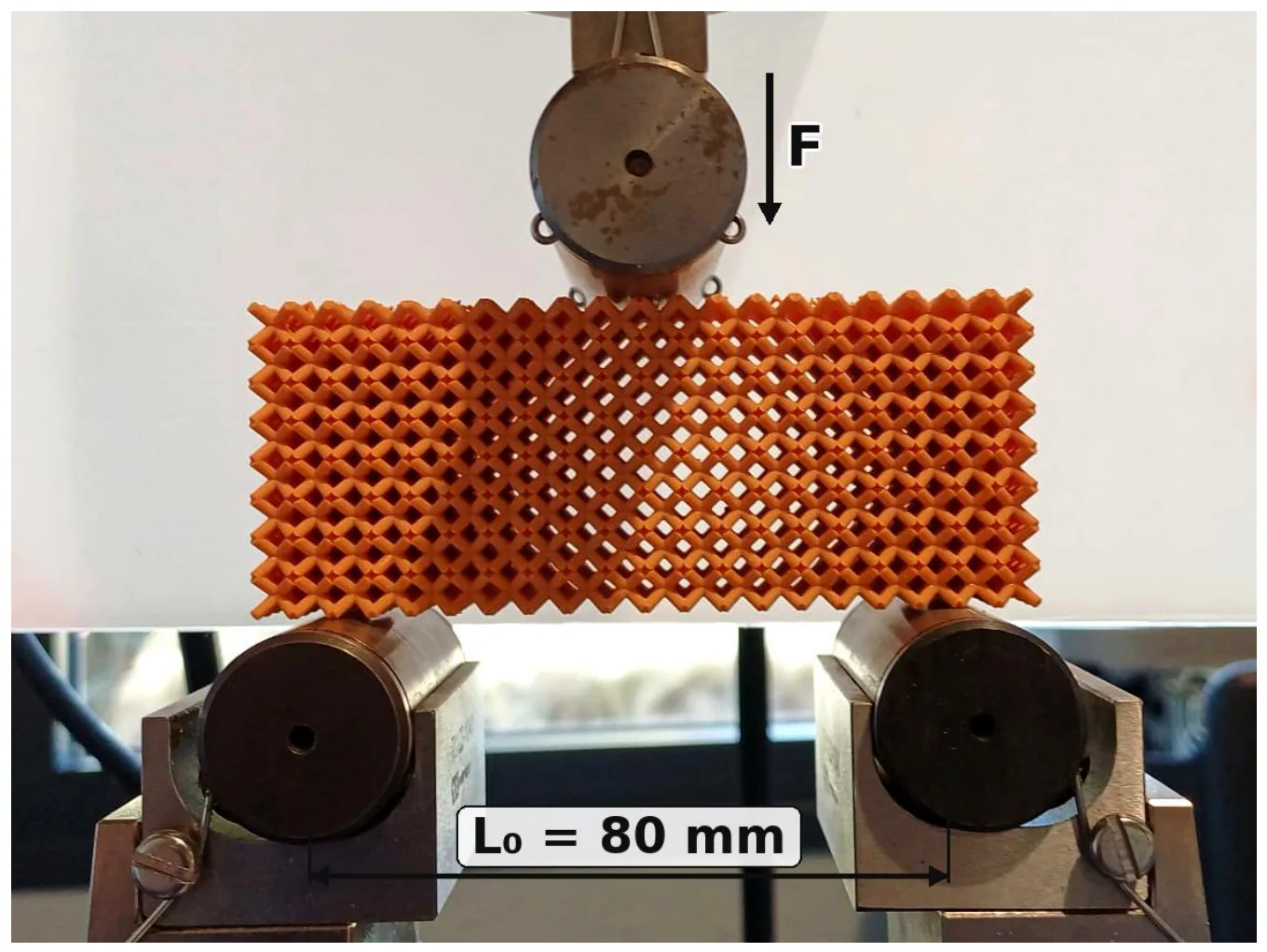
Real three-point bending test setup on the Instron 8801 frame. The BCC lattice beam rests on two cylindrical support rollers spanning $L_{0}=80$ mm, while the load *F* is applied at mid-span through the upper roller (r=12.5 mm).

The beams were subjected to three-point bending tests with a support span of $L_{0}=80$ mm throughout the experiments, as shown in Figure 6 and Figure 7. The overall beam length was kept constant at 100 mm in all cases. The radius of the loading and support rollers was r=12.5 mm.

The lattice architecture was defined by limiting the number of unit cells across the beam width to four unit cells. The beam height was varied considering 4, 6, and 8 unit cells in the vertical direction, allowing the influence of the structural height *h* to be investigated while maintaining a constant beam length. The experiments were carried out with the same test machine previously described, although the load cell was swapped by a 5 kN cell to increase the precision of the results. Each specimen was tested a minimum of three times at a speed of 1 mm/min under ambient laboratory conditions of 20–25 °C.

The vertical displacement *u* at the loading point was recorded directly from the crosshead of the testing machine. Two factors support the use of this measurement as an estimate of the mid-span deflection of the specimens. First, the bending loads involved were modest (of the order of tens to a few hundred newtons) and were applied through a stiff servo-hydraulic frame equipped with a 5 kN load cell, so that the elastic compliance of the machine is expected to be small compared with the deflection of the beams. Second, despite their cellular nature, the specimens are comparatively stiff owing to their high relative density ($\rho^{*}\approx 0.423$), and the loading and support rollers, of radius r=12.5 mm, distribute the contact force over several surface struts. Nevertheless, local contact compliance at the roller–lattice interface cannot be ruled out from the present measurements and is therefore considered as one of the possible contributors to the apparent additional compliance discussed below.

To quantify these systematic contributions, a Hertzian line-contact estimate was made for the steel roller (r=12.5 mm, $E_{1}\approx 200$ GPa, $\nu_{1}=0.30$) in contact with a solid-resin half-space ($E_{s}=1724.7$ MPa, $\nu_{s}=0.35$), giving a plane-strain effective modulus $E^{*}\approx 1948$ MPa. For the present load range this yields a sub-millimetre contact width (2a≈0.4–0.7 mm), peak pressures of 16–26 MPa and a local indentation of only a few micrometres, i.e., two to three orders of magnitude below the measured deflections. Similarly, accounting for the additional compliance of the deepest beams (at most ≈$2\times 10^{-3}$ mm/N, see Section 4.2.1) by machine compliance alone would require a load-train stiffness of only ∼500 N/mm, far below that of the servo-hydraulic frame used here. Machine and solid-material contact compliance are therefore small contributors; because they were not measured independently, however, local roller–lattice compliance is retained as one of the possible mechanisms contributing to the apparent additional compliance discussed below, rather than being ruled out entirely.

**Figure 8:**
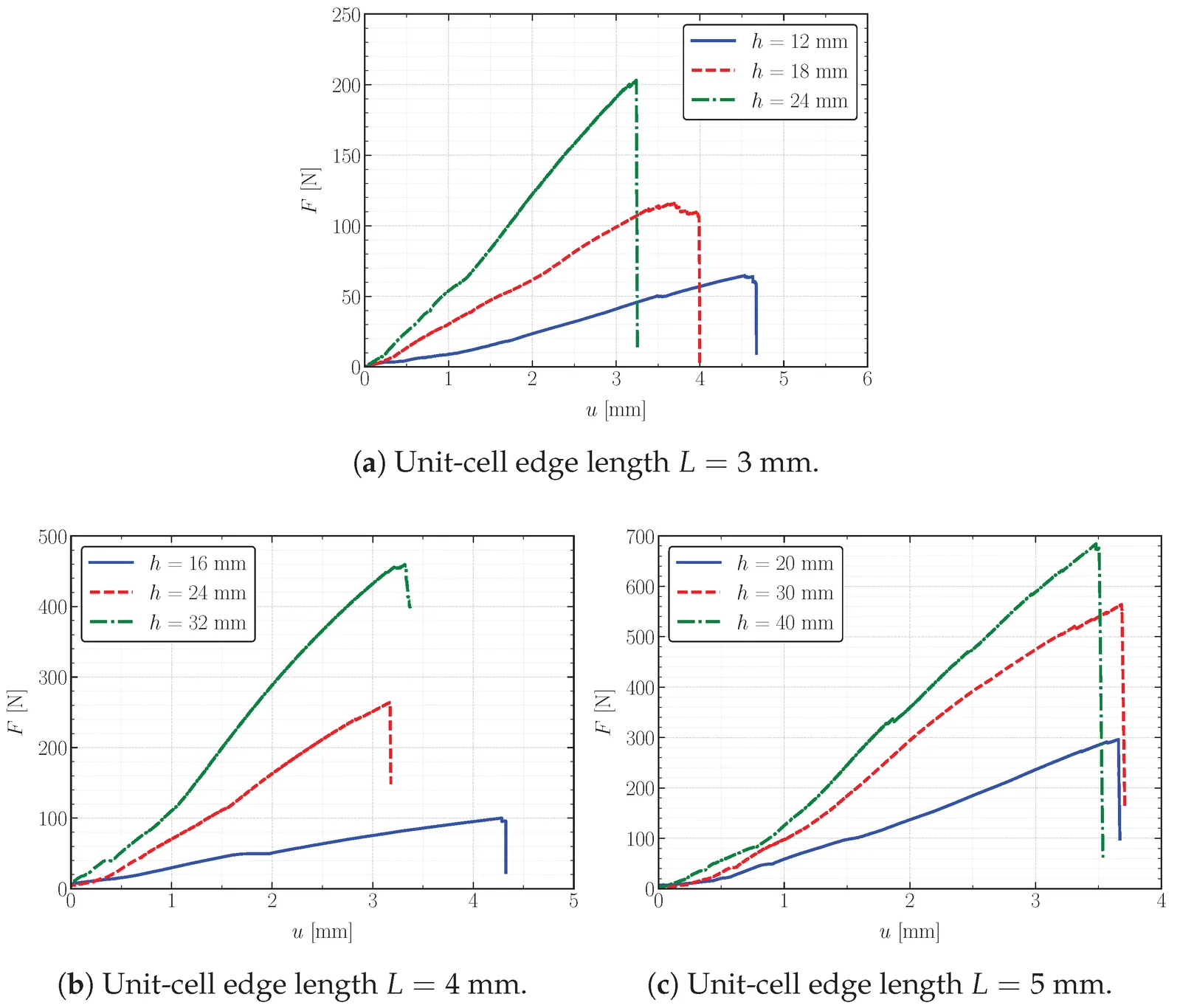
Representative force–displacement curves from three-point bending tests for each unit-cell edge length considered: (**a**) L=3 mm, (**b**) L=4 mm, and (**c**) L=5 mm. In all cases, results are shown for beam heights of 4, 6, and 8 unit cells. Increasing beam height *h* produces higher stiffness and failure load, while the displacement at failure decreases accordingly.

Representative force–displacement curves obtained from the three-point bending tests are presented in Figure 8 for unit-cell edge lengths L=3, 4, and 5 mm. In each case, results are shown for beam heights corresponding to 4, 6, and 8 unit cells in the vertical direction. As expected, an increase in beam height *h* leads to a notable increase in both the initial stiffness and the failure load. Conversely, the displacement at failure decreases with increasing *h*, which is consistent with the higher stress concentrations induced in stiffer configurations. Failure occurred in a brittle manner in all tested configurations: upon reaching the maximum load, a sudden and abrupt drop in the recorded force was observed, with no evidence of a progressive or ductile post-peak response. In every specimen, fracture initiated at mid-span on the lower (tensile) face of the beam and propagated upwards through the lattice. This consistent failure location indicates that tensile stresses on the bottom fibers govern the ultimate response, which is also in line with the brittle nature of the photopolymer resin and justifies the abrupt load drop observed across all unit-cell sizes and beam heights considered in this study. The initiation on the tension side, together with the single-event load drop and the absence of any progressive roll-off, is characteristic of tensile fracture of the outer struts and nodal junctions on the bottom fibre, rather than of elastic buckling of compressive struts, which would be expected to produce a more gradual post-peak response. This is consistent with the non-slender, node-dominated character of the present struts (R/L=1/6, $\rho^{*}\approx 0.423$).

## 3. Analytical Models

In this section, the analytical models employed to predict the mechanical behavior of the BCC lattice structures are presented. First, two expressions for the effective Young’s modulus are introduced. The model of Tancogne-Dejean and Mohr [25] is formulated in terms of the closest-neighbor distance *ℓ*, whereas the expression of Lee et al. [26] is evaluated using the cubic unit-cell dimensions. The distinction between *L* and *ℓ* is therefore retained throughout the analysis. Subsequently, the bending rigidity of the homogenized beam is derived using a classical Euler–Bernoulli beam model, a BCC-specific shear-corrected homogenized beam model, and a strain-gradient Euler–Bernoulli extension. The shear-corrected model uses an analytical BCC shear modulus at the unit-cell level and introduces a finite-section shear-efficiency factor to account for the discrete shear-transfer mechanism of the lattice beam.

### 3.1. Effective Young’s Modulus

#### 3.1.1. Model Based on Tancogne-Dejean and Mohr [25]

Tancogne-Dejean and Mohr [25] derived closed-form expressions for the homogenized elastic moduli of BCC truss lattices. In their formulation, the characteristic length *ℓ* corresponds to the closest-neighbor distance. Therefore, the relevant geometrical parameter for this model is

(5) $$\xi_{\mathrm{TD}}=\frac{R}{\ell }.$$

For beams of uniform circular cross-section and negligible shear deformation at the strut level, the Euler–Bernoulli expression for the Young’s modulus in the [100] direction can be written as

(6) $${\bar{E}}_{\mathrm{TD},\mathrm{EB}}=\frac{9\sqrt{3}\pi }{2}\left(\frac{1}{1-\sqrt{2}\xi_{\mathrm{TD}}}\right)\left(\frac{1}{1-2\sqrt{2}\xi_{\mathrm{TD}}+\frac{7}{2}\xi_{\mathrm{TD}}^{2}}\right)\xi_{\mathrm{TD}}^{4}E_{s}.$$

Because the present struts are relatively stocky, the Timoshenko-corrected version of the model was also evaluated following the stiffness-based formulation given by Tancogne-Dejean and Mohr. The details of this calculation, including the evaluation of $K_{a}$, $K_{f}$, the nodal correction factor γ, and the Timoshenko correction factor $C_{T}$, are provided in Appendix A.

#### 3.1.2. Model Based on Lee et al. [26]

An alternative expression for the effective Young’s modulus of BCC lattice structures was proposed by Lee et al. [26], based on a free-body diagram analysis of the BCC unit cell subjected to compressive loading. The displacement of the unit cell is decomposed into axial and bending contributions of the individual struts following Euler–Bernoulli beam theory. For a cubic unit cell with *L* and circular cross-section of radius *R*, the effective Young’s modulus reads

(7) $${\bar{E}}_{\mathrm{Lee}}=\frac{8\pi \sqrt{3}\;\xi_{\mathrm{Lee}}^{4}\;E_{s}}{2\xi_{\mathrm{Lee}}^{2}+1},\;\xi_{\mathrm{Lee}}=\frac{R}{L}.$$

Unlike the model of Tancogne-Dejean and Mohr, this expression is based on a direct free-body analysis of the cubic BCC unit cell and does not explicitly introduce a nodal-volume correction. In the present work, it is retained as a compact analytical estimate directly expressed in terms of the cubic unit-cell dimensions.

It is worth noting that the analytical approach adopted by Lee et al. [26] is based on the methodology originally proposed by Ptochos and Labeas [27], who derived closed-form expressions for the effective elastic modulus of BCC micro-lattice structures by decomposing the strut deformation into axial and bending contributions within the framework of Euler–Bernoulli beam theory. The model of Lee et al. follows the same theoretical foundation and, for the cubic unit-cell case considered in the present work, yields predictions that differ only marginally from those of Ptochos and Labeas. For this reason, only the expression proposed by Lee et al. is retained in the following bending comparisons, as it provides a compact closed-form expression directly applicable to the BCC unit-cell geometry considered here.

### 3.2. Bending Rigidity

#### 3.2.1. Classical Euler–Bernoulli Beam Model

The classical Euler–Bernoulli solution gives the maximum mid-span deflection under three-point bending as

(8) $$w_{EB}=\frac{PL_{0}^{3}}{48\bar{E}\bar{I}},$$

where $L_{0}$ is the support span, *P* is the applied load, $\bar{E}$ is the effective Young’s modulus, and $\bar{I}$ is the homogenized second moment of area. The latter is approximated using the outer dimensions of the lattice beam as

(9) $$\bar{I}=\frac{bh^{3}}{12},$$

where *b* is the width and *h* is the height of the homogenized rectangular cross-section. The corresponding homogenized cross-sectional area is

(10) $$\bar{A}=bh.$$

The cross-sectional dimensions and the corresponding values of $\bar{A}$ and $\bar{I}$ for each specimen configuration are listed in Table 2.

The bending stiffness, defined as D=P/w, can then be written as

(11) $$D_{EB}=\frac{48\bar{E}\bar{I}}{L_{0}^{3}}.$$

#### 3.2.2. Shear-Corrected Homogenized Beam Model

The classical Euler–Bernoulli formulation assumes that the transverse deflection is dominated by bending and neglects shear deformation. This approximation is appropriate for slender beams, but it may become inaccurate when the span-to-depth ratio decreases. In the present experimental campaign, the support span was kept constant at $L_{0}=80\;\mathrm{mm}$, whereas the beam height varied from h=12 to 40mm. Therefore, the ratio $L_{0}/h$ ranges from 6.67 for the most slender configuration to 2.00 for the deepest beam. For the latter configurations, shear flexibility at the structural beam level may contribute significantly to the measured displacement.

To account for this effect, a shear-corrected homogenized beam model is introduced. The total mid-span deflection under three-point bending is written as the sum of a bending contribution and a shear contribution,

(12) $$w_{T}=\frac{PL_{0}^{3}}{48\bar{E}\bar{I}}+\frac{PL_{0}}{4\kappa_{\mathrm{BCC}}{\bar{G}}_{\mathrm{BCC}}\bar{A}},$$

where $\bar{E}$ is the effective Young’s modulus of the BCC lattice, $\bar{I}$ and $\bar{A}$ are the gross second moment of area and cross-sectional area of the homogenized beam, ${\bar{G}}_{\mathrm{BCC}}$ is the effective shear modulus of the BCC unit cell, and $\kappa_{\mathrm{BCC}}$ is a finite-section shear-efficiency factor for the BCC lattice beam.

The corresponding initial stiffness is

(13) $$D_{T}=\frac{P}{w_{T}}={\left[\frac{L_{0}^{3}}{48\bar{E}\bar{I}}+\frac{L_{0}}{4\kappa_{\mathrm{BCC}}{\bar{G}}_{\mathrm{BCC}}\bar{A}}\right]}^{-1}.$$

Equivalently,

(14) $$D_{T}=\eta_{T}D_{EB},$$

with

(15) $$\eta_{T}={\left[1+\frac{\bar{E}}{{\bar{G}}_{\mathrm{BCC}}}\frac{h^{2}}{\kappa_{\mathrm{BCC}}L_{0}^{2}}\right]}^{-1}.$$

This form shows explicitly that the shear correction becomes relevant as $h/L_{0}$ increases. For slender beams, $h/L_{0}\ll 1$, and therefore $\eta_{T}\to 1$, recovering the Euler–Bernoulli prediction. For deeper beams, $\eta_{T}<1$, and the effective stiffness is reduced by the additional shear compliance.

The classical value κ=5/6 is the shear correction factor commonly associated with a homogeneous rectangular section, obtained from an energy-equivalent representation of the non-uniform shear stress distribution. A BCC lattice beam, however, is not a homogeneous solid rectangular section. Its shear response is governed by a finite number of inclined struts, discrete nodal regions, non-periodic boundary conditions, and local perturbations introduced by the loading and support rollers. Therefore, the rectangular-section value is not adopted directly as the final shear correction factor.

The effective shear modulus of the BCC unit cell is instead estimated from the analytical expression proposed for regular BCC open-cell structures by Ptochos and Labeas,

(16) $${\bar{G}}_{\mathrm{BCC}}=\frac{32\pi R^{4}E_{s}+16\pi R^{2}E_{s}L^{2}}{12\sqrt{3}L^{4}}.$$

For the present geometry, with R/L=1/6 and $E_{s}=1724.7\;\mathrm{MPa}$, this gives

(17) $${\bar{G}}_{\mathrm{BCC}}\approx 122.3\;\mathrm{MPa}.$$

This value should be interpreted as a unit-cell-level homogenized shear modulus over the gross cell volume.

Since the finite lattice beam does not transmit shear through a continuous rectangular solid area, an additional section-efficiency factor is introduced. As a first-order, semi-empirical estimate—not derived from a rigorous energy-equivalence analysis of the discrete BCC section and not claimed to be a transferable material property—this factor is taken as

(18) $$\kappa_{\mathrm{BCC}}=\frac{\rho^{*}}{3}.$$

The factor $\rho^{*}$ accounts for the fact that only a fraction of the gross cross-section is occupied by solid material, while the factor 1/3 provides a simple orientation-efficiency estimate associated with the three-dimensional inclined strut network. For the present specimens, $\rho^{*}\approx 0.423$, and therefore

(19) $$\kappa_{\mathrm{BCC}}\approx 0.141.$$

It is important to emphasize that Equation (18) is not introduced as an independently validated material parameter. Rather, it is used as a compact engineering estimate of the apparent shear-transfer efficiency of a finite BCC lattice section. It collects, in an average sense, the fact that the section is discrete, that shear is transmitted through a finite number of inclined struts and nodal regions, and that boundary cells and roller-contact perturbations may alter the local deformation field. The shear-corrected model is therefore interpreted as a semi-empirical, diagnostic description of the additional compliance expected in deep beams, not as a universal predictive theory for all lattice geometries. Its adequacy is assessed a posteriori through the sensitivity analysis reported in Section 4.2, which shows that $\kappa_{\mathrm{BCC}}=\rho^{*}/3$ lies close to the value that minimises the global prediction error for the present dataset.

#### 3.2.3. Strain-Gradient Euler–Bernoulli Beam Model

In the scope of the present work, we also consider the strain-gradient beam theory presented in [23,28,29]. The deflection for the Euler–Bernoulli beam within the strain-gradient framework is given by

(20) $$w_{gr}=\frac{PL_{0}^{3}}{48\left(\bar{E}\bar{I}+\bar{E}\bar{A}g^{2}\right)},$$

where *g* is the intrinsic length scale parameter, which acts as a higher-order material parameter depending on the microstructure of the unit cell. This parameter characterises size-dependent beam behaviour, in which smaller beams may exhibit a stiffening effect relative to the prediction of classical theory. The corresponding bending stiffness reads

(21) $$D_{gr}=D_{EB}\left[1+12{\left(\frac{g}{h}\right)}^{2}\right].$$

The additional term $12{\left(g/h\right)}^{2}$ reflects the contribution of the microstructural length scale to the overall bending resistance and directly implies that $D_{gr}>D_{EB}$ for any nonzero value of *g*. Consequently, the strain-gradient formulation consistently predicts higher bending stiffnesses than the classical theory, with the difference becoming more pronounced as the beam height *h* decreases relative to *g*.

It should therefore be noted that this correction can only increase the stiffness predicted by the classical Euler–Bernoulli model. Consequently, it cannot improve configurations for which the classical model already overestimates the experimental stiffness. In the present work, *g* is calibrated from the bending data for each unit-cell edge length in order to assess whether such a correction can improve the predictions. However, the resulting values and their effect on the model accuracy are discussed critically in the Results section. As shown there, this comparison is reported as a negative result: because the strain-gradient term is strictly stiffening ($D_{gr}\geq D_{EB}$), it is the wrong degree of freedom for the deep, dense beams that dominate the present discrepancies, and it does not contradict its established usefulness for slender, stiffening-dominated lattices reported elsewhere.

## 4. Results and Discussion

This section presents a comprehensive comparison of the experimental and analytical results obtained for the BCC lattice specimens considered in this study. The analysis is organized into two parts: uniaxial compression results and three-point bending results. For each case, the predictions derived from the analytical models are contrasted against the experimentally measured data. The predictive capability of each model is evaluated in terms of initial stiffness. Special attention is given to the influence of the unit cell edge length *L*, the beam height *h*, and the span-to-depth ratio $L_{0}/h$ on the mechanical response, as well as to the limitations of each modelling approach in capturing the observed behaviour.

### 4.1. Compression Results

The mean values and standard deviations of the effective Young’s modulus obtained from the three compression tests performed for each unit-cell size are presented in Table 3, together with the analytical predictions derived from the models of Tancogne-Dejean and Mohr [25] and Lee et al. [26]. The standard deviation values are in all cases below 10% of the mean, which is considered a reasonable level of dispersion for this type of experimental campaign and demonstrates the good repeatability of the results. Reporting 95% confidence intervals (Student’s *t*, n=3), the effective moduli are 54.01±12.12, 50.01±4.97 and 50.60±10.96 MPa for L=3, 4 and 5 mm, respectively; the relatively wide intervals reflect the small sample size rather than poor repeatability. Three replicates were tested for each configuration, consistent with the bending campaign; this limited number is acknowledged as a limitation.

As can be observed, the model of Lee et al. provides predictions in close agreement with the experimental results, with discrepancies below approximately 10% for all unit-cell sizes. In contrast, the Euler–Bernoulli version of the Tancogne-Dejean and Mohr model significantly overestimates the effective Young’s modulus. When the Timoshenko correction at the strut level is included, the prediction is reduced from 135.99 MPa to 87.85 MPa. Although this correction substantially improves the prediction, the model still remains stiffer than the experimental response. This discrepancy may be associated with the assumptions of ideal periodicity, rigid nodes, affine nodal motion, and perfect strut geometry, which are particularly strong for additively manufactured polymeric specimens.

Regarding maximum compressive stress, the mean values obtained experimentally are 2.34±0.15 MPa, 1.95±0.02 MPa, and 1.94±0.27 MPa for unit-cell edge lengths L=3, 4, and 5 mm, respectively. A slight decrease in failure stress is observed with an increase in unit-cell size, which is consistent with the trend observed for the effective Young’s modulus and reflects the influence of the structural scale on the failure behavior of the lattice specimens.

As shown in this section, the model proposed by Lee et al. [26] provides excellent agreement with the experimental results in terms of effective Young’s modulus prediction. For this reason, the bending stiffness predictions presented in the following section adopt the effective modulus obtained from the model of Lee et al., Equation (7), as input to the homogenized beam models.

### 4.2. Bending Results

**Figure 9:**
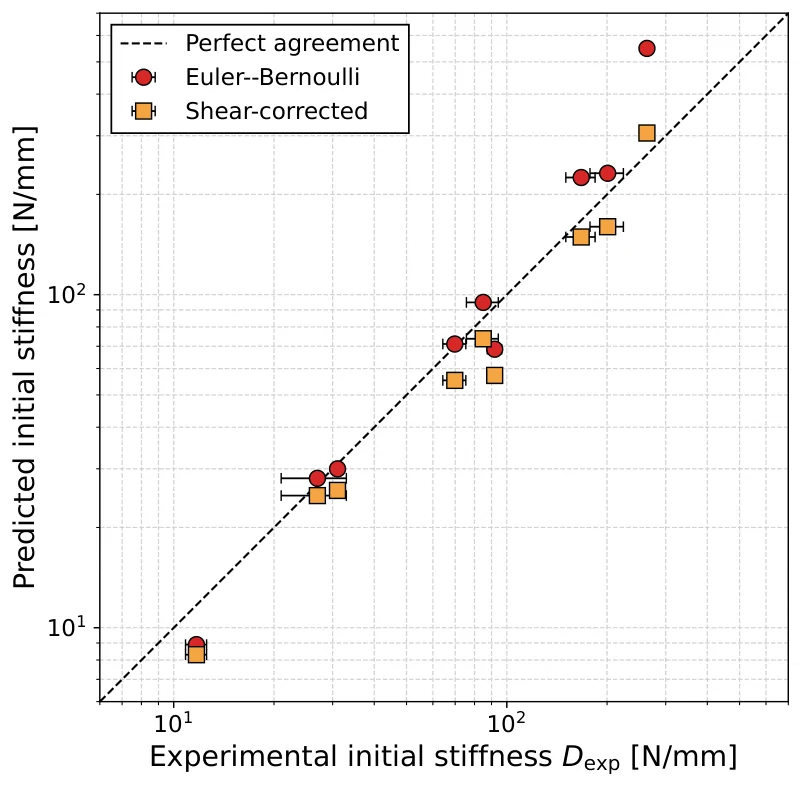
Predicted versus experimental initial stiffness for the nine configurations reported in Table 4. Horizontal error bars denote the experimental standard deviation, and the dashed line indicates perfect agreement. The largest deviation corresponds to the Euler–Bernoulli prediction for the deepest beam (L=5 mm, h=40 mm), which the shear-corrected model brings substantially closer to the experimental value.

The mean values of initial stiffness obtained from the three-point bending tests are presented in Table 4, together with the corresponding standard deviations, the predictions of the classical Euler–Bernoulli model, and those of the BCC shear-corrected homogenized beam model evaluated using Equations (13)–(19). As expected, the initial stiffness increases monotonically with beam height *h* for all unit-cell sizes considered. The standard deviation values are generally small relative to the mean, indicating good repeatability across the three tests performed for each configuration. The coefficient of variation is below about 12% in almost all cases (the exception being L=4 mm, h=16 mm, at 22%), and 95% confidence intervals (Student’s *t*, n=3) were computed for every configuration. The comparison between measured and predicted stiffness is summarized graphically in Figure 9, in which departures from the dashed diagonal directly indicate prediction errors.

The results show that the classical Euler–Bernoulli model provides predictions of acceptable accuracy for several configurations considered, with errors below approximately 16% in five out of nine cases. However, significant discrepancies are observed in specific configurations. The largest errors occur for L=3 mm with the shortest beam (h=12 mm, error of 24.0%), for L=4 mm with the tallest beam (h=32 mm, error of 34.4%), and for L=5 mm with the tallest beam (h=40 mm, error of 108.3%). It is also worth noting that the classical model does not exhibit a consistent bias: in some configurations it underestimates the experimental stiffness, while in others it overestimates it, as is apparent from the distribution of points on both sides of the diagonal in Figure 9. This suggests that the discrepancies are not solely attributable to a systematic limitation of the homogenization approach, but rather to the combined influence of shear flexibility, boundary effects, discrete lattice architecture, and the ratio between the beam height and the span length.

The BCC shear correction has a minor effect for the most slender specimens, but becomes increasingly relevant as the span-to-depth ratio decreases. As shown in Table 4 and Figure 9, the correction substantially reduces the overprediction for the deepest beams, at the cost of introducing additional flexibility in configurations for which the Euler–Bernoulli prediction was already accurate.

**Figure 10:**
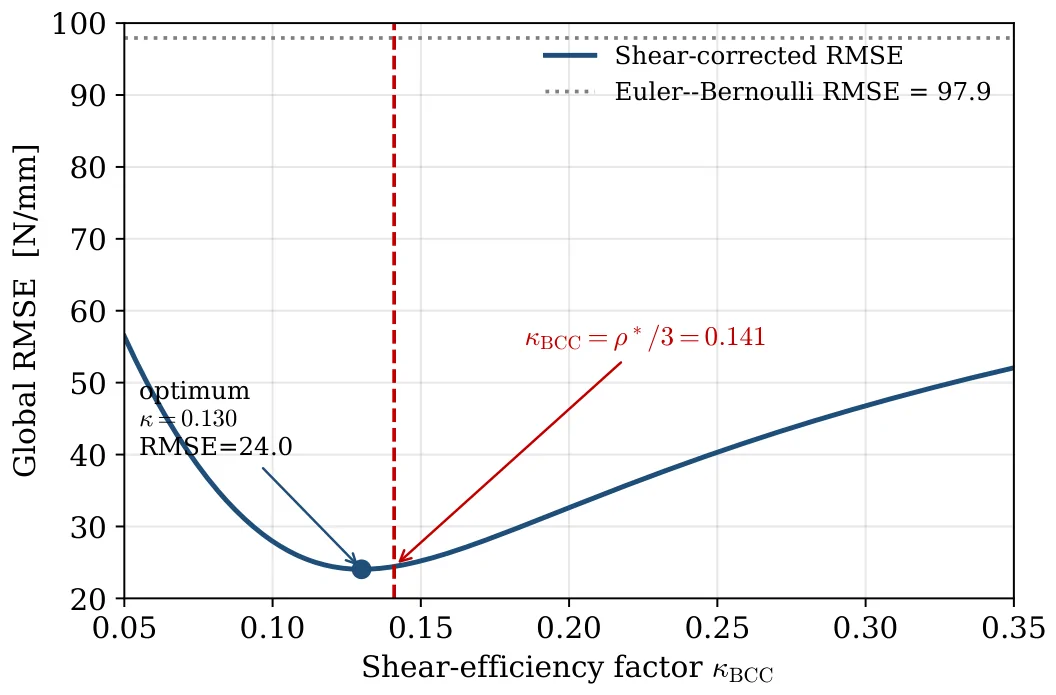
Global RMSE of the shear-corrected homogenized beam model as a function of the shear-efficiency factor $\kappa_{\mathrm{BCC}}$. The physically motivated value $\kappa_{\mathrm{BCC}}=\rho^{*}/3=0.141$ (dashed line) lies on the flat minimum, close to the RMSE-optimal value κ≈0.130; the dotted line marks the Euler–Bernoulli RMSE.

Because the shear-efficiency factor $\kappa_{\mathrm{BCC}}=\rho^{*}/3$ is a first-order engineering estimate, its effect on the predictions was examined through a sensitivity analysis of the global root-mean-square error (RMSE) over a physically plausible range of $\kappa_{\mathrm{BCC}}$, with all other quantities fixed (Figure 10 and Table 5). The RMSE exhibits a shallow minimum: the RMSE-optimal value is κ≈0.130 (RMSE =24.05 N/mm), while the physically motivated estimate $\rho^{*}/3=0.141$ gives RMSE =24.43 N/mm. The two differ by less than 8% in κ and by less than 2% in RMSE, so $\rho^{*}/3$ lies essentially on the flat optimum; the agreement of the shear-corrected model in the deep-beam regime is therefore not an artefact of a fortuitous choice of κ.

#### 4.2.1. Experimental Compliance Decomposition

To avoid attributing all discrepancies to a single mechanism, the bending data were further analyzed in terms of compliance rather than stiffness. The experimental and Euler–Bernoulli compliances are defined as

(22) $$C_{\exp}=\frac{1}{D_{\exp}},\;C_{\mathrm{EB}}=\frac{1}{D_{\mathrm{EB}}},$$

and the apparent non-Euler–Bernoulli contribution is written as

(23) $$C_{\mathrm{add}}=C_{\exp}-C_{\mathrm{EB}}.$$

A normalized measure of this additional compliance is therefore

(24) $$\Delta_{C}=\frac{C_{\exp}-C_{\mathrm{EB}}}{C_{\mathrm{EB}}}=\frac{D_{\mathrm{EB}}}{D_{\exp}}-1.$$

This quantity is useful because shear deformation, contact compliance, and local deformation mechanisms can only add compliance to the Euler–Bernoulli bending contribution. Therefore, configurations with $\Delta_{C}>0$ are more compliant than predicted by the classical homogenized beam model, whereas configurations with $\Delta_{C}<0$ are experimentally stiffer than the Euler–Bernoulli prediction and cannot be explained by shear flexibility alone.

The resulting compliance-decomposition values are reported in Table 6.

For the configurations with $C_{\mathrm{add}}>0$, an apparent efficiency factor can be obtained by equating the measured additional compliance to a Timoshenko-like shear term,

(25) $$C_{\mathrm{add}}\approx \frac{L_{0}}{4\kappa_{\mathrm{app}}{\bar{G}}_{\mathrm{BCC}}\bar{A}},$$

which gives

(26) $$\kappa_{\mathrm{app}}=\frac{L_{0}}{4{\bar{G}}_{\mathrm{BCC}}\bar{A}C_{\mathrm{add}}}.$$

The resulting values vary substantially across the tested geometries, from values above unity when the additional compliance is nearly negligible to approximately 0.10 for the deepest beam. This wide spread is expected rather than contradictory: $\kappa_{\mathrm{app}}$ is obtained by dividing the span-related term by $C_{\mathrm{add}}$, so for the near-Euler–Bernoulli configurations $C_{\mathrm{add}}\to 0$ the quotient becomes ill-conditioned and its large values (e.g., 2.17) are numerical artefacts of a vanishing denominator, not physical efficiencies. The physically meaningful values are those of the deep-beam regime, where $C_{\mathrm{add}}$ is significant and $\kappa_{\mathrm{app}}$ settles to ≈0.10–0.21, bracketing $\kappa_{\mathrm{BCC}}=0.141$. This confirms that a single constant shear-efficiency factor cannot be interpreted as a universal property of the lattice material. Instead, $\kappa_{\mathrm{app}}$ should be understood as an apparent parameter that collects structural shear, local contact compliance, finite-cell effects, nodal-region deformation, and the discrete distribution of load paths across the section.

The BCC shear-corrected model substantially reduces the overprediction observed for the deepest beams. The most notable case is the configuration L=5mm, h=40mm, for which the Euler–Bernoulli model predicts an initial stiffness of 548.8N/mm, whereas the experimental value is 263.47N/mm. Including the BCC shear correction reduces the prediction to 305.69N/mm, decreasing the error from 108.3% to 16.0%. A similar improvement is observed for L=4mm, h=32mm, where the error decreases from 34.4% to 10.9%.

However, the shear correction does not improve all configurations. For the L=3mm series, the Euler–Bernoulli model already provides accurate predictions for h=18 and 24mm. In these cases, the additional shear flexibility introduced by the BCC correction leads to an underestimation of the experimental stiffness. The compliance decomposition clarifies this behavior: several slender or intermediate configurations have $\Delta_{C}\approx 0$ or even $\Delta_{C}<0$, meaning that there is little apparent additional compliance to be corrected, or that the experiment is stiffer than the Euler–Bernoulli estimate. Such cases cannot be explained by structural shear alone. They likely reflect the combined influence of discrete cross-sectional architecture, finite-cell geometry, nodal-region morphology, and the fact that the gross-section inertia used in the homogenized beam model is only an idealized representation of the actual lattice section.

Overall, the comparison suggests that Euler–Bernoulli theory is adequate for the configurations in which the apparent additional compliance remains small, whereas the BCC shear-corrected model becomes more appropriate for the deepest beams with low $L_{0}/h$ ratios. Therefore, the two models should not be interpreted as competing universal descriptions. The complementary character of the two formulations can be described through a single dimensionless parameter. From Equation (12), the ratio of the shear to the bending compliance is

(27) $$\chi =\frac{\bar{E}}{\kappa_{\mathrm{BCC}}{\bar{G}}_{\mathrm{BCC}}}{\left(\frac{h}{L_{0}}\right)}^{2},$$

which is precisely the term entering $\eta_{T}={\left(1+\chi \right)}^{-1}$ in Equation (15). Shear flexibility ceases to be a minor perturbation once it contributes a comparable fraction of the total compliance; adopting χ≈0.5 as a practical diagnostic threshold, the BCC shear-corrected model is preferable for χ≳0.5 and the classical Euler–Bernoulli model otherwise. For the present lattice, $\bar{E}/\left(\kappa_{\mathrm{BCC}}{\bar{G}}_{\mathrm{BCC}}\right)\approx 3.18$, so this threshold corresponds to $L_{0}/h\approx 2.5$. This criterion identifies precisely the deep configurations for which the shear-corrected model gives the lower prediction error ($L_{0}/h=2.5$ and 2.0). The threshold should be regarded as indicative, since it was established from nine configurations at a single relative density and strut-to-cell ratio; its transferable form is the compliance ratio χ rather than the bare span-to-depth ratio. No confidence interval can be attached to it from the present data, and the numerical value $L_{0}/h\approx 2.5$ applies only to lattices with an $\bar{E}/\left(\kappa_{\mathrm{BCC}}{\bar{G}}_{\mathrm{BCC}}\right)$ ratio comparable to that of the present specimens; it should not be read as a general design rule.

#### 4.2.2. Role of Non-Slender Struts, Nodes, and Finite-Cell Effects

The compliance analysis also shows why the observed discrepancies should not be assigned exclusively to shear deformation. The present specimens have R/L=1/6 and $\rho^{*}\approx 0.423$, so the struts are not slender in the asymptotic sense assumed by many truss-based homogenized expressions, and the nodal regions represent a non-negligible fraction of the solid phase. Under bending, these nodal regions are not merely passive volume corrections: they affect the rotational stiffness of strut junctions, the transfer of shear across the section, and the way boundary cells interact with the loading and support rollers.

Moreover, the beam cross-section contains only four unit cells across the width and four to eight unit cells through the height. The use of the gross rectangular second moment of area in Equation (9) is therefore a homogenized approximation to a strongly discrete distribution of load-bearing struts. Depending on the relative position of the outer struts, boundary nodes, and roller contact regions, this approximation may either overestimate or underestimate the actual bending stiffness. This explains why the sign of $\Delta_{C}$ is not uniform across the dataset. Positive values of $\Delta_{C}$ indicate missing compliance relative to the Euler–Bernoulli model, whereas negative values indicate that the experimental structure is stiffer than the gross-section homogenized estimate. The latter cases cannot be corrected by adding a Timoshenko-like shear term, because such a term only increases compliance. The same sign argument excludes machine and solid-material roller-contact compliance as the origin of the negative $\Delta_{C}$ values: both can only add compliance, and their magnitude was shown in Section 2.3 to be small (a few micrometres of Hertzian indentation, and a machine contribution below ∼5% of the deep-beam $C_{\mathrm{add}}$). A negative $\Delta_{C}$ must therefore originate from the idealisation of the cross-section itself—the gross-section second moment of area over- or under-estimating the true load-bearing inertia of the discrete boundary cells—rather than from shear, contact, or machine effects.

Consequently, the shear-corrected model is best interpreted as a low-order representation of the deep-beam regime rather than as a complete physical decomposition. The present experiments support the conclusion that deep dense BCC lattice beams require additional non-Euler–Bernoulli compliance, but the data alone do not uniquely separate structural shear from nodal deformation, contact compliance, and finite-cell effects. Fully discriminating these mechanisms requires dedicated discrete-section or micropolar lattice-beam simulations; the purpose of the present study is to provide a controlled experimental benchmark and a model-independent compliance decomposition that such refined descriptions must reproduce.

**Figure 11:**
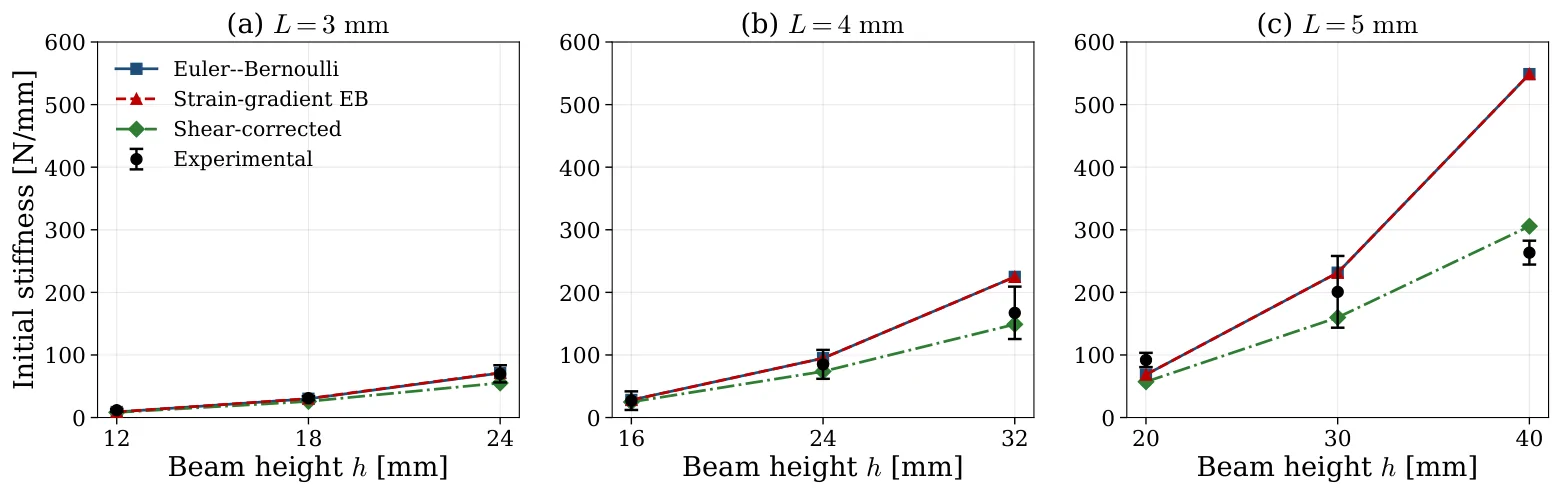
Comparison of experimental initial stiffness and analytical predictions from the Euler–Bernoulli, strain-gradient Euler–Bernoulli, and shear-corrected homogenized beam models for unit-cell edge lengths (**a**) L=3 mm, (**b**) L=4 mm, and (**c**) L=5 mm. Error bars on the experimental data denote 95% confidence intervals (Student’s *t*, n=3). The strain-gradient parameter was calibrated as g=0.561 mm for L=3 mm, and g=0 for L=4 and L=5 mm.

The initial stiffness predictions obtained from the Euler–Bernoulli, strain-gradient Euler–Bernoulli, and shear-corrected homogenized beam models are compared against the experimental results in Figure 11. For the strain-gradient model, the intrinsic length scale parameter *g* was determined for each unit-cell edge length by minimizing the root mean square error with respect to the experimental data. The calibrated values obtained are g=0.561 mm for L=3 mm, and g=0 mm for both L=4 mm and L=5 mm. The latter result implies that the strain-gradient correction vanishes for the two larger unit-cell sizes, and both models therefore yield identical predictions in those cases.

The intrinsic length parameter *g* was calibrated independently for each unit-cell edge length. For L=3 mm, the calibrated value g=0.561 mm produces only a minor modification of the classical Euler–Bernoulli prediction, because *g* remains much smaller than the beam heights considered. The maximum correction occurs for h=12 mm and is approximately 2.6%. For L=4 and 5 mm, the optimal value is g=0, and the strain-gradient model therefore collapses to the classical Euler–Bernoulli prediction. This result is expected because the adopted strain-gradient correction is strictly stiffening, whereas the largest discrepancies occur in configurations for which the classical model already overestimates the experimental stiffness. This confirms that the strain-gradient correction does not provide a significant improvement for the present dataset.

Table 7 presents the root mean square error (RMSE) of the initial stiffness predictions obtained from the Euler–Bernoulli, strain-gradient Euler–Bernoulli, and shear-corrected homogenized beam models.

The RMSE values confirm that the strain-gradient correction does not significantly modify the Euler–Bernoulli predictions for the present dataset. In contrast, the BCC shear-corrected model substantially reduces the global RMSE, from 97.94 to 24.44N/mm. This improvement is mainly associated with the deepest beams, for which the span-to-depth ratio is low and the compliance decomposition indicates a large positive $\Delta_{C}$. Nevertheless, the model worsens the predictions for L=3mm, where the Euler–Bernoulli approximation was already accurate or slightly conservative. This behavior reinforces the regime-dependent interpretation: Euler–Bernoulli theory is more suitable when the apparent additional compliance is small, whereas the BCC shear-corrected formulation is more appropriate as a diagnostic approximation for deep lattice beams in which $C_{\mathrm{add}}$ becomes significant.

The maximum loads recorded during the three-point bending tests are presented in Table 8. As observed for the initial stiffness, the maximum load increases monotonically with beam height *h* for all unit-cell sizes. The standard deviation values are in most cases below 15% of the mean, reflecting acceptable repeatability, with the exception of the configuration L=3 mm, h=18 mm, which exhibits a somewhat larger dispersion likely associated with the inherent variability of the failure process in brittle lattice structures.

For a fixed unit-cell size, the maximum load scales strongly with beam height, which is consistent with the increased cross-sectional resistance of taller specimens. Comparing across unit-cell sizes at equivalent beam heights, larger unit cells tend to sustain higher failure loads, which can be attributed to the greater strut radius used in those configurations to maintain a constant R/L. As discussed previously, failure occurs in a brittle manner in all cases, with an abrupt load drop upon reaching $F_{\max}$, and no evidence of progressive post-peak response.

It is worth noting that, given the brittle failure observed in all configurations and the absence of a progressive post-peak response, the energy absorbed up to peak load is essentially governed by the initial stiffness and the displacement at failure. Consequently, integrating the force–displacement curves does not provide additional physical insight beyond the initial stiffness and maximum load values already reported in Table 4 and Table 8.

## 5. Conclusions

This work presented a combined experimental and analytical study of the flexural behaviour of polymeric BCC lattice beams fabricated by low-force stereolithography. Nine beam configurations, spanning three unit-cell edge lengths (L=3,4,5 mm) at a constant strut-to-cell ratio R/L=1/6 ($\rho^{*}\approx 0.423$), were tested under three-point bending, with the effective Young’s modulus independently characterized by uniaxial compression on cubic specimens. Three homogenized frameworks were assessed: a classical Euler–Bernoulli model and a BCC shear-corrected model, both evaluated without calibration to the bending data, and a strain-gradient extension whose intrinsic length scale was calibrated against them.

For the effective modulus, the closed-form expression of Lee et al. [26] reproduced the compression data within approximately 10%, whereas the Tancogne-Dejean and Mohr model [25] remained markedly stiffer even after the strut-level Timoshenko correction. The former was therefore adopted as input to the bending models. This agreement should nevertheless be interpreted with caution: strut-based analytical models are nominally restricted to idealized slender-strut lattices, whereas the present specimens lie in a dense regime in which nodal regions and non-slender struts are expected to influence the mechanical response.

In bending, no single homogenized model proved adequate over the full geometric range. The Euler–Bernoulli formulation performed well for several configurations but overestimated the stiffness by more than 100% for the deepest specimen. The shear-corrected model, based on the BCC unit-cell shear modulus ${\bar{G}}_{\mathrm{BCC}}$ and the first-order finite-section efficiency estimate $\kappa_{\mathrm{BCC}}=\rho^{*}/3$, brought the deep-beam predictions within approximately 16% and reduced the global RMSE from 97.94 to 24.44N/mm. However, it introduced excessive flexibility in configurations for which the Euler–Bernoulli model was already accurate. The shear-corrected formulation should therefore not be interpreted as a universal predictive theory, but as a low-order diagnostic correction for the deep-beam regime.

A compliance decomposition was introduced to make this interpretation explicit. The normalized additional compliance $\Delta_{C}=\left(C_{\exp}-C_{\mathrm{EB}}\right)/C_{\mathrm{EB}}$ was close to zero or negative in several slender and intermediate configurations, showing that the discrepancy cannot be attributed to shear flexibility alone. In contrast, $\Delta_{C}$ increased strongly for the deepest beams, reaching 1.083 for L=5mm and h=40mm, meaning that the experimental compliance was more than twice the Euler–Bernoulli bending compliance. An apparent efficiency factor $\kappa_{\mathrm{app}}$ was also extracted for the cases with positive additional compliance. Its strong variation across the dataset confirms that the apparent extra compliance collects several mechanisms, including structural shear, local roller-contact compliance, finite-cell effects, nodal-region deformation, and the discrete distribution of struts across the section.

The two beam descriptions are therefore complementary rather than competing. Their range of applicability can be described through the shear-to-bending compliance ratio $\chi =\left(\bar{E}/\kappa_{\mathrm{BCC}}{\bar{G}}_{\mathrm{BCC}}\right){\left(h/L_{0}\right)}^{2}$: the shear-corrected model is preferable for χ≳0.5 and the Euler–Bernoulli model otherwise, a threshold that for the present lattice corresponds to $L_{0}/h\approx 2.5$. This criterion should be regarded as indicative, since it was established from nine configurations at a single relative density and strut-to-cell ratio. The strain-gradient extension yielded no appreciable improvement: being strictly stiffening, it cannot recover configurations in which the classical model already overpredicts the response.

Taken together, these findings delineate the range of validity of simple homogenized beam models for dense finite BCC lattice structures. The main conclusion is not that a single missing correction explains the experiments, but rather that the flexural response results from the combined influence of structural shear, non-slender struts, nodal-region morphology, finite-cell and boundary effects, local roller-contact compliance, and the discrete cross-sectional distribution of struts. The dataset and compliance-based interpretation reported here are intended to support the development and validation of more refined descriptions, such as discrete-section or micro-polar lattice beam formulations, together with independent future measurements or simulations of shear and contact effects.

## Figures and Tables

**Table 1:** Printing and post-processing parameters (Raise3D DF2, Apricot V1 resin).

Parameter	Value
Slicing software	ideaMaker v5.2.4.8581
Process template	0.05 mm–Light–DF2–High Detail Apricot V1
Layer thickness	0.050 mm
Number of base layers	3
Normal-layer exposure (contour/fill)	4.0/9.0 s
Base-layer exposure (contour/fill)	60.0/60.0 s
Support exposure (normal/base)	9.0/60.0 s
Wait before exposure (normal/base)	2.5/60.0 s
LED power mode	2
Anti-aliasing	grey range, 0–255
In-plane scale compensation (*X*, *Y*)	100.69%
Contour (edge) offset	−0.054 mm
Slow/fast lift distance (normal)	1.5/7.0 mm
Slow/fast lift speed (normal)	1.0/6.0 mm s^−1^
Chamber temperature (preheat)	25 °C (5 min)
Build orientation	flat; layers horizontal, load ⊥ layers
Support type	touch-platform-only, $40^{\circ }$ overhang
Support point spacing (edge)	2.5 mm (1.5 mm)
Washing time	10 min
UV post-curing (DF-Cure)	15 min per side
Heat-cure temperature	60 °C

**Table 2:** Homogenized cross-sectional properties—effective area $\bar{A}$ and second moment of area $\bar{I}$—of the BCC lattice beam specimens as a function of unit-cell edge length *L* and beam height *h*.

*L*	*b*	*h*	$\bar{A}$	$\bar{I}$
[mm]	[mm]	[mm]	[mm^2^]	[mm^4^]
	12.0	12.0	144.0	1728.0
3.0	12.0	18.0	216.0	5832.0
	12.0	24.0	288.0	13,824.0
	16.0	16.0	256.0	5461.3
4.0	16.0	24.0	384.0	18,432.0
	16.0	32.0	512.0	43,690.7
	20.0	20.0	400.0	13,333.3
5.0	20.0	30.0	600.0	45,000.0
	20.0	40.0	800.0	106,666.7

**Table 3:** Comparison of experimental and analytical predictions of the effective Young’s modulus for each cubic unit-cell edge length *L*.

*L*	Experimental	Lee et al.	TD–EB	TD–Timoshenko
[mm]	[MPa]	[MPa]	[MPa]	[MPa]
3.0	54.01±4.88	54.88	135.99	87.85
4.0	50.01±2.00	54.88	135.99	87.85
5.0	50.60±4.41	54.88	135.99	87.85

**Table 4:** Experimental initial stiffness (mean ± standard deviation) obtained from the three-point bending tests, together with the predictions of the classical Euler–Bernoulli model and of the BCC shear-corrected homogenized beam model. Percentage errors are computed with respect to the experimental mean value.

*L*	*h*	$L_{0}/h$	Experimental	EB	EB Error	Shear-Corrected	Shear Error
[mm]	[mm]	[-]	[N/mm]	[N/mm]	[%]	[N/mm]	[%]
	12.0	6.67	11.70±0.85	8.9	24.0	8.30	29.1
3.0	18.0	4.44	31.03±1.27	30.0	3.3	25.84	16.7
	24.0	3.33	69.80±5.53	71.1	1.9	55.29	20.8
	16.0	5.00	27.00±5.98	28.1	4.1	24.93	7.7
4.0	24.0	3.33	84.97±9.32	94.8	11.6	73.73	13.2
	32.0	2.50	167.27±16.85	224.8	34.4	148.97	10.9
	20.0	4.00	91.97±4.59	68.6	25.4	57.22	37.8
5.0	30.0	2.67	200.83±23.06	231.5	15.3	159.96	20.3
	40.0	2.00	263.47±7.66	548.8	108.3	305.69	16.0

**Table 5:** Global RMSE of the shear-corrected model as a function of the shear-efficiency factor $\kappa_{\mathrm{BCC}}$ (all other parameters fixed). The Euler–Bernoulli RMSE is 97.9 N/mm.

$\kappa_{\mathrm{BCC}}$	0.10	0.12	0.130 ^†^	0.141 ^‡^	0.16	0.20	0.30	0.50
RMSE [N/mm]	27.9	24.4	24.05	24.43	26.4	32.6	46.8	63.1

^†^ RMSE-optimal value. ^‡^ physically motivated value $\rho^{*}/3$.

**Table 6:** Experimental compliance decomposition relative to the Euler–Bernoulli prediction. The apparent efficiency factor $\kappa_{\mathrm{app}}$ is reported only for configurations with $C_{\mathrm{add}}>0$ and should be interpreted as an apparent shear/contact/finite-size efficiency, not as an independently measured shear correction factor.

*L*	*h*	$L_{0}/h$	$\Delta_{C}$	$\kappa_{\mathrm{app}}$
[mm]	[mm]	[-]	[-]	[-]
	12.0	6.67	−0.239	–
3.0	18.0	4.44	−0.033	–
	24.0	3.33	0.019	2.17
	16.0	5.00	0.041	0.44
4.0	24.0	3.33	0.116	0.35
	32.0	2.50	0.344	0.21
	20.0	4.00	−0.254	–
5.0	30.0	2.67	0.153	0.41
	40.0	2.00	1.083	0.10

**Table 7:** Root mean square error (RMSE) of the initial stiffness predictions obtained from the Euler–Bernoulli, strain-gradient Euler–Bernoulli, and BCC shear-corrected homogenized beam models. The global RMSE is computed using all nine bending configurations.

*L*	Euler–Bernoulli	Strain-Gradient EB	Shear-Corrected
[mm]	[N/mm]	[N/mm]	[N/mm]
3.0	1.89	1.85	9.11
4.0	33.70	33.70	12.46
5.0	166.24	166.24	39.41
Global	97.94	97.94	24.44

**Table 8:** Mean values and standard deviations of the maximum load obtained from the three-point bending tests, as a function of unit-cell edge length *L* and beam height *h*.

*L*	*h*	$F_{\max}$
[mm]	[mm]	[N]
	12.0	61.50±2.78
3.0	18.0	114.70±22.35
	24.0	213.83±12.37
	16.0	111.33±10.06
4.0	24.0	253.60±18.85
	32.0	429.50±54.53
	20.0	274.03±24.39
5.0	30.0	545.10±35.92
	40.0	675.57±17.76

## Data Availability

The data presented in this study are available on request from the corresponding author.

## Appendix A. Details of the Analytical Evaluation of the Effective Young’s Modulus

This appendix summarizes the analytical calculations used to evaluate the effective Young’s modulus of the BCC lattice specimens. Two models are considered: the expression proposed by Lee et al. [26], and the stiffness-based formulation of Tancogne-Dejean and Mohr [25]. Particular attention is paid to the distinction between the cubic unit-cell edge length and the closest-neighbor distance, since this distinction affects the evaluation of both the relative density and the Tancogne-Dejean and Mohr model.

### Appendix A.1. Geometrical Definitions

The specimens considered in this work are based on a cubic BCC unit cell of edge length *L*. The struts connect the center of the cube to its eight vertices. Therefore, the center-to-vertex distance, also referred to as the closest-neighbor distance, is

(A1) $$\ell =\frac{\sqrt{3}}{2}L.$$

In the present specimens, the strut radius was scaled with the unit-cell edge length, such that

(A2) $$\frac{R}{L}=\frac{1}{6}\approx 0.1667.$$

The geometrical values used in the calculations are summarized in Table A1.

**Table A1:** Geometrical parameters used for the analytical evaluation of the BCC lattice specimens. Here, *L* denotes the cubic unit-cell edge length and *ℓ* denotes the center-to-vertex strut length.

*L*	*R*	*ℓ*	R/L	R/ℓ
[mm]	[mm]	[mm]	[-]	[-]
3.0	0.500	2.598	0.1667	0.1925
4.0	0.667	3.464	0.1667	0.1925
5.0	0.833	4.330	0.1667	0.1925

It is useful to emphasize the geometrical convention adopted in the present work. Here, *L* denotes the edge length of the cubic BCC unit cell, consistently with the notation used in the specimen drawings and experimental description. The center-to-vertex distance, or closest-neighbor distance, is denoted by *ℓ*. The analytical formulation of Tancogne-Dejean and Mohr is evaluated in terms of this closest-neighbor distance. Therefore, when their model is applied to the present specimens, the relevant geometrical parameter is not R/L, but

(A3) $$\xi_{\mathrm{TD}}=\frac{R}{\ell }=\frac{R}{(\sqrt{3}/2)L}=\frac{2}{\sqrt{3}}\frac{R}{L}.$$

For the present geometry, R/L=1/6, and therefore

(A4) $$\xi_{\mathrm{TD}}=\frac{R}{\ell }\approx 0.1925.$$

On the other hand, the model of Lee et al. is directly expressed in terms of the cubic unit-cell dimensions. Consequently, the relevant geometrical parameter for that model is

(A5) $$\xi_{\mathrm{Lee}}=\frac{R}{L}.$$

This distinction is retained throughout the calculations below.

### Appendix A.2. Relative Density

For a BCC lattice made of uniform circular struts, Tancogne-Dejean and Mohr [25] express the relative density as

(A6) $$\rho^{*}=3\sqrt{3}\pi {\left(\frac{R}{\ell }\right)}^{2}-18\sqrt{2}{\left(\frac{R}{\ell }\right)}^{3}.$$

Using $\xi_{\mathrm{TD}}=R/\ell$, this can be written as

(A7) $$\rho^{*}=3\sqrt{3}\pi \xi_{\mathrm{TD}}^{2}-18\sqrt{2}\xi_{\mathrm{TD}}^{3}.$$

For L=4mm and R=0.667mm, for example,

(A8) $$\ell =\frac{\sqrt{3}}{2}\left(4\right)=3.464\;\mathrm{mm},$$

(A9) $$\xi_{\mathrm{TD}}=\frac{0.667}{3.464}=0.1925,$$

and therefore

(A10) $$\rho^{*}=3\sqrt{3}\pi {\left(0.1925\right)}^{2}-18\sqrt{2}{\left(0.1925\right)}^{3}=0.423.$$

The resulting relative density is the same for the three unit-cell sizes because R/L was kept constant. The values are shown in Table A2.

**Table A2:** Relative density obtained using the geometrical convention of Tancogne-Dejean and Mohr [25].

*L*	R/ℓ	$\rho^{*}$
[mm]	[-]	[-]
3.0	0.1925	0.423
4.0	0.1925	0.423
5.0	0.1925	0.423

### Appendix A.3. Effective Young’s Modulus According to Lee et al. [26]

Lee et al. [26] derived an analytical expression for the effective Young’s modulus of a BCC lattice under compressive loading by decomposing the strut displacement into axial and bending contributions. For the cubic case considered here, the expression can be written as

(A11) $${\bar{E}}_{\mathrm{Lee}}=\frac{8\pi \sqrt{3}\xi_{\mathrm{Lee}}^{4}E_{s}}{2\xi_{\mathrm{Lee}}^{2}+1},$$

where

(A12) $$\xi_{\mathrm{Lee}}=\frac{R}{L},$$

and $E_{s}$ is the Young’s modulus of the base material. In this work,

(A13) $$E_{s}=1724.7\;\mathrm{MPa}.$$

Since R/L=1/6 for all configurations,

(A14) $$\xi_{\mathrm{Lee}}=\frac{1}{6}=0.1667.$$

Substituting into Equation (A11),

(A15) $${\bar{E}}_{\mathrm{Lee}}=\frac{8\pi \sqrt{3}{\left(0.1667\right)}^{4}\left(1724.7\right)}{2{\left(0.1667\right)}^{2}+1}=54.88\;\mathrm{MPa}.$$

The values obtained for all specimens are summarized in Table A3.

**Table A3:** Effective Young’s modulus predicted by the model of Lee et al. [26].

*L*	*R*	R/L	${\bar{E}}_{\mathrm{Lee}}$
[mm]	[mm]	[-]	[MPa]
3.0	0.500	0.1667	54.88
4.0	0.667	0.1667	54.88
5.0	0.833	0.1667	54.88

Since R/L is constant for the three configurations, Equation (A11) predicts the same effective Young’s modulus, ${\bar{E}}_{\mathrm{Lee}}=54.88\;\mathrm{MPa}$, for all unit-cell sizes. This value is adopted in the bending calculations reported in the main text.

### Appendix A.4. Effective Young’s Modulus According to Tancogne-Dejean and Mohr [25]

The model of Tancogne-Dejean and Mohr [25] is formulated in terms of the axial stiffness $K_{a}$ and the effective flexural stiffness $K_{f}$ of a doubly-clamped beam embedded in the BCC network. The effective Young’s modulus in the [100] direction can be written as

(A16) $${\bar{E}}_{\mathrm{TD}}=\frac{3\sqrt{3}}{2}\frac{K_{f}}{\ell }\frac{1}{1+\frac{1}{2}\frac{K_{f}}{K_{a}}}.$$

In this formulation, the free length of the deformable part of the strut is reduced by the presence of rigid nodal regions. This is accounted for through

(A17) $$\ell_{\mathrm{eff}}=\gamma \ell ,$$

with

(A18) $$\gamma =1-\sqrt{2}\frac{R}{\ell }.$$

For uniform circular struts, the axial stiffness is

(A19) $$K_{a}=\frac{\pi E_{s}R^{2}}{\ell }\frac{1}{\gamma }.$$

The Euler–Bernoulli flexural stiffness is

(A20) $$K_{f,\mathrm{EB}}=\frac{3\pi E_{s}R^{4}}{\ell^{3}}\frac{1}{\gamma^{3}}.$$

For relatively stocky beams, Tancogne-Dejean and Mohr also introduce a Timoshenko correction through the factor $C_{T}$, such that

(A21) $$K_{f,\mathrm{Tim}}=C_{T}K_{f,\mathrm{EB}},$$

where

(A22) $$C_{T}=\frac{1}{1+\left(\frac{7+12\nu_{s}+4\nu_{s}^{2}}{1+\nu_{s}}\right){\left(\frac{R}{\ell }\right)}^{2}\frac{1}{\gamma^{2}}}.$$

The Poisson’s ratio of the base material is taken as

(A23) $$\nu_{s}=0.35.$$

For L=4mm, R=0.667mm, and ℓ=3.464mm, the nodal correction factor is

(A24) $$\gamma =1-\sqrt{2}\left(0.1925\right)=0.7278.$$

The axial stiffness is therefore

(A25) $$K_{a}=\frac{\pi \left(1724.7\right){\left(0.667\right)}^{2}}{3.464}\frac{1}{0.7278}=955.12\;N/\mathrm{mm}.$$

The Euler–Bernoulli flexural stiffness is

(A26) $$K_{f,\mathrm{EB}}=\frac{3\pi \left(1724.7\right){\left(0.667\right)}^{4}}{{\left(3.464\right)}^{3}}\frac{1}{{\left(0.7278\right)}^{3}}=200.33\;N/\mathrm{mm}.$$

Thus,

(A27) $$\frac{K_{f,\mathrm{EB}}}{K_{a}}=\frac{200.33}{955.12}=0.210.$$

Substitution into Equation (A16) gives

(A28) $${\bar{E}}_{\mathrm{TD},\mathrm{EB}}=\frac{3\sqrt{3}}{2}\frac{200.33}{3.464}\frac{1}{1+\frac{1}{2}\left(0.210\right)}=135.99\;\mathrm{MPa}.$$

For the Timoshenko correction, the shear correction factor is

(A29) $$C_{T}=\frac{1}{1+\left(\frac{7+12\left(0.35\right)+4{\left(0.35\right)}^{2}}{1+0.35}\right){\left(0.1925\right)}^{2}\frac{1}{{\left(0.7278\right)}^{2}}}=0.623.$$

Therefore,

(A30) $$K_{f,\mathrm{Tim}}=0.623\left(200.33\right)=124.79\;N/\mathrm{mm}.$$

and

(A31) $$\frac{K_{f,\mathrm{Tim}}}{K_{a}}=\frac{124.79}{955.12}=0.131.$$

The Timoshenko-corrected effective Young’s modulus is then

(A32) $${\bar{E}}_{\mathrm{TD},\mathrm{Tim}}=\frac{3\sqrt{3}}{2}\frac{124.79}{3.464}\frac{1}{1+\frac{1}{2}\left(0.131\right)}=87.85\;\mathrm{MPa}.$$

The complete set of values is given in Table A4.

**Table A4:** Intermediate quantities and effective Young’s modulus predicted by the Tancogne-Dejean and Mohr model.

*L*	*R*	γ	$K_{a}$	$K_{f,\mathrm{EB}}$	$C_{T}$	${\bar{E}}_{\mathrm{TD},\mathrm{EB}}$	${\bar{E}}_{\mathrm{TD},\mathrm{Tim}}$
[mm]	[mm]	[-]	[N/mm]	[N/mm]	[-]	[MPa]	[MPa]
3.0	0.500	0.7278	716.34	150.25	0.623	135.99	87.85
4.0	0.667	0.7278	955.12	200.33	0.623	135.99	87.85
5.0	0.833	0.7278	1193.90	250.41	0.623	135.99	87.85

### Appendix A.5. Effective Shear Modulus and Finite-Section Shear Efficiency

The shear-corrected beam model used in the main text requires an effective shear modulus and a shear-efficiency factor for the finite BCC lattice beam. The effective shear modulus is evaluated using the analytical expression proposed by Ptochos and Labeas [27] for regular BCC open-cell structures,

(A33) $${\bar{G}}_{\mathrm{BCC}}=\frac{32\pi R^{4}E_{s}+16\pi R^{2}E_{s}L^{2}}{12\sqrt{3}L^{4}}.$$

For the present geometry, R/L=1/6 and $E_{s}=1724.7\;\mathrm{MPa}$. Since the ratio R/L is constant, Equation (A33) gives the same value for all three unit-cell sizes,

(A34) $${\bar{G}}_{\mathrm{BCC}}=122.3\;\mathrm{MPa}.$$

The finite-section shear-efficiency factor is taken as

(A35) $$\kappa_{\mathrm{BCC}}=\frac{\rho^{*}}{3}.$$

For $\rho^{*}\approx 0.423$, this gives

(A36) $$\kappa_{\mathrm{BCC}}=0.141.$$

The product $\kappa_{\mathrm{BCC}}{\bar{G}}_{\mathrm{BCC}}$ is the effective shear rigidity per unit gross area entering the structural-level shear compliance. For the present specimens,

(A37) $$\kappa_{\mathrm{BCC}}{\bar{G}}_{\mathrm{BCC}}=17.25\;\mathrm{MPa}.$$

This parameter is not introduced as a second homogenization of the relative density. Rather, ${\bar{G}}_{\mathrm{BCC}}$ represents the unit-cell-level homogenized shear modulus, while $\kappa_{\mathrm{BCC}}$ approximates the finite-section efficiency of shear transfer in a lattice beam with a limited number of cells through the cross-section.

### Appendix A.6. Comparison of Analytical Predictions

The remaining discrepancy between the Timoshenko-corrected Tancogne-Dejean and Mohr model and the experimental data may be associated with the assumptions of ideal periodicity, rigid nodes, affine deformation of the nodal centers, and perfect strut geometry. These assumptions are restrictive for additively manufactured polymeric specimens, especially at the relatively high relative density considered here, where nodal regions represent a significant fraction of the solid volume.
